# Expression Atlas update: from tissues to single cells

**DOI:** 10.1093/nar/gkz947

**Published:** 2019-10-30

**Authors:** Irene Papatheodorou, Pablo Moreno, Jonathan Manning, Alfonso Muñoz-Pomer Fuentes, Nancy George, Silvie Fexova, Nuno A Fonseca, Anja Füllgrabe, Matthew Green, Ni Huang, Laura Huerta, Haider Iqbal, Monica Jianu, Suhaib Mohammed, Lingyun Zhao, Andrew F Jarnuczak, Simon Jupp, John Marioni, Kerstin Meyer, Robert Petryszak, Cesar Augusto Prada Medina, Carlos Talavera-López, Sarah Teichmann, Juan Antonio Vizcaino, Alvis Brazma

**Affiliations:** 1 European Molecular Biology Laboratory, European Bioinformatics Institute, EMBL-EBI, Hinxton, UK; 2 Wellcome Sanger Institute, Wellcome Genome Campus, Hinxton CB10 1SA, UK; 3 Cancer Research UK Cambridge Institute, University of Cambridge, Cambridge, UK

## Abstract

Expression Atlas is EMBL-EBI’s resource for gene and protein expression. It sources and compiles data on the abundance and localisation of RNA and proteins in various biological systems and contexts and provides open access to this data for the research community. With the increased availability of single cell RNA-Seq datasets in the public archives, we have now extended Expression Atlas with a new added-value service to display gene expression in single cells. Single Cell Expression Atlas was launched in 2018 and currently includes 123 single cell RNA-Seq studies from 12 species. The website can be searched by genes within or across species to reveal experiments, tissues and cell types where this gene is expressed or under which conditions it is a marker gene. Within each study, cells can be visualized using a pre-calculated t-SNE plot and can be coloured by different features or by cell clusters based on gene expression. Within each experiment, there are links to downloadable files, such as RNA quantification matrices, clustering results, reports on protocols and associated metadata, such as assigned cell types.

## INTRODUCTION

Expression Atlas (https://www.ebi.ac.uk/gxa/home) is an added-value online bioinformatics resource that includes a database, user interface and web-service enabling easy access to information about gene expression across species, tissues, cells, experimental conditions and diseases. It was originally developed in 2009 (1) as a resource to uniformly analyse, annotate and display the results of microarray and RNA-Seq experiments from the ArrayExpress archive (2). Since then, Expression Atlas has grown to process datasets from a variety of other sources and archives, such as NCBI’s Gene Expression Omnibus (GEO) (3), the European Nucleotide Archive (4) as well as controlled access datasets, such as GTEx. In addition, Expression Atlas serves as a long term portal for transcriptomics data generated by large-scale genomics studies such as Gramene (5), the comparative resource for plants (http://www.gramene.org/) and the Pancancer Analysis of Whole Genomes (PCAWG, https://docs.icgc.org/pcawg/). Expression Atlas has also integrated protein expression information via mass spectrometry proteomics datasets coming from the PRIDE database (6), and one dataset from the Human Protein Atlas. Over the last five years, processed and curated gene expression datasets have been regularly contributed to projects such as Open Targets (7), whilst Expression Atlas’ heatmap widget has been adopted by eight resources to display gene expression across different tissues within their websites (e.g. Reactome (8)).

With recent advances in technologies for tissue dissociation and high-throughput sequencing at the single cell level, single cell RNA-Seq datasets are being generated and deposited into the public domain at an increasing pace. Since the last Expression Atlas update in 2017 (9), the main developments within Expression Atlas have therefore focused on the annotation, analysis, storage and display of single cell RNA-Seq studies. The Expression Atlas, previously consisting of a ‘baseline expression’ and a ‘differential expression’ component for expression at the level of tissues, has now acquired a third, ‘single cell’ component. This new component, the Single Cell Expression Atlas, was first launched in 2018 and currently includes 123 studies, including almost a million assays from 12 different species. Single Cell Expression Atlas employs analysis pipelines that process datasets from SMART-like and droplet-based experimental protocols in a standardised way and displays gene expression in different cells and cell types.

**Figure 1.**
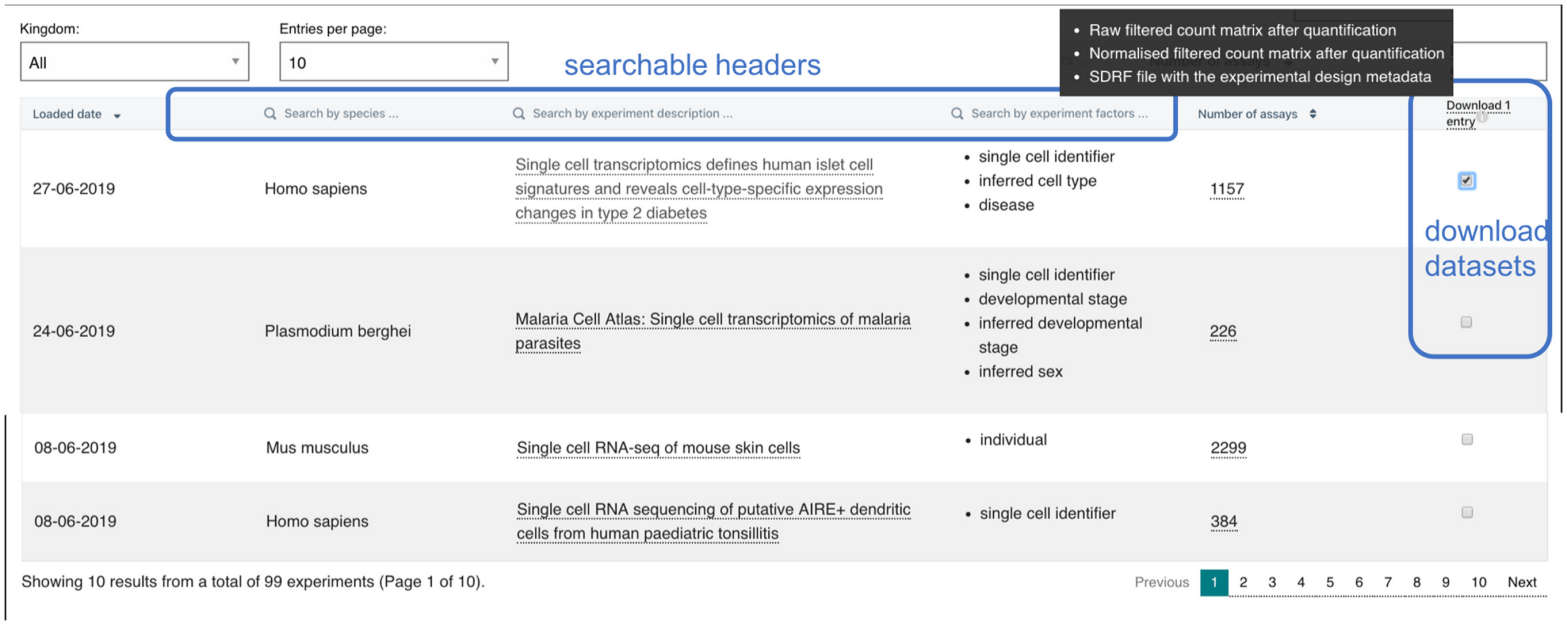
Screenshot of the ‘Browse Experiments’ page. A user can search through the experiments by species or other keywords, quickly link to their results pages or download raw, normalized data and metadata bundles from one or more studies.

Single Cell Expression Atlas is unique in that it provides to the life sciences community uniformly analysed and annotated single cell RNA-Seq data across multiple species. It enables easy access to meaningful results and standardised data files (see Figure 1), therefore providing easy input to software for further analysis gene of expression matrices, as well as supporting the development of computational methods for downstream analyses, such as cell type annotation. The field of single cell genomics is undergoing rapid developments in the range of experimental protocols. These result in large increases in data volumes, stressing the underlying infrastructure and causing frequent rounds of re-engineering. For example, bulk RNA-Seq experiments in Expression Atlas consist of just above hundred thousand assays across 809 studies, whereas 123 single cell studies comprise almost a million assays. Moreover, new analysis methods are constantly being generated and significantly improved, resulting in re-evaluations and updates of pipelines. Therefore, Single Cell Expression Atlas was launched to reside as a service within Expression Atlas with the expectation to be fully integrated and queried via Expression Atlas’ main gene and condition search in the near future, as the experimental protocols and analysis methods mature.

## SINGLE CELL EXPRESSION ATLAS

### Data

Since its original launch in May 2018, the Single Cell Expression Atlas service has processed 123 studies consisting of 955,000 assays, from 12 different species, including *Homo sapiens* and model organisms such as *Mus musculus*, *Arabidopsis thaliana* and *Drosophila melanogaster*. All datasets are sourced from public archives, such as ArrayExpress and GEO. Metadata are curated in-house through a semi-automatic process of identifying the experimental factors, such as cell types, diseases or perturbations, followed by annotation with Experimental Factor Ontology terms (EFO) to describe the experimental comparisons for further processing. Samples are annotated with cell types, where these have been submitted or made available by the primary authors of these datasets. Cell types are then linked with terms from the Cell Ontology.

### Data analysis

Analysis pipelines for Single Cell Expression Atlas comprise two parts: gene expression quantification and downstream analysis. Whilst quantification is accomplished using technology-dependent pipelines, downstream analysis is currently performed identically for all studies.

#### Quantification of gene expression

For SMART-like technologies (10) where data are available in cell-demultiplexed form, a bulk-like pipeline is used. This pipeline was originally implemented in iRAP (https://doi.org/10.1101/005991), but has recently been re-implemented using Nextflow (11). The pipeline comprises quality filtering, quality trimming and sequencing artifact removal with FASTX-Toolkit (http://hannonlab.cshl.edu/fastx_toolkit/), poly-A and uncalled base filtering with fastq-utils (https://github.com/nunofonseca/fastq_utils) and a contamination check. Paired-end reads are re-paired with fastq-pair (https://doi.org/10.1101/552885) after filtering. Filtered reads are then quantified with Kallisto (12), and cell-wise quantifications subsequently combined to a final matrix.

For droplet technologies such as 10x (v2, v3) (13) and Drop-seq (14) with cell barcodes and unique molecular identifiers (UMIs), we employ Alevin, which is part of the Salmon package (15). Alevin handles barcode/UMI processing, quantification and the production of a quantification matrix. By default, Alevin filters cell barcodes based on a frequency distribution, but we found this difficult to operate in an unsupervised manner over many experiments. We therefore run Alevin without filtering and excluding only the lowest frequency barcodes, and subsequently apply the emptyDrops method of the DropletUtils package (16) to remove empty droplets. Where a study combines multiple libraries, the matrices resulting from each run are combined into a final matrix. This pipeline is under close review, with processes such as doublet removal being considered for inclusion in future iterations.

#### Downstream analysis

For all single cell experiments for downstream analysis we use Scanpy (17), including additional filtering, dimension reduction, clustering and marker detection. Final output files are provided in standard file formats where available, including a 10x-like matrix market format for expression matrices. We envisage use of formats allowing richer data (e.g. Loom (http://loompy.org/) or annData (17)), the inclusion of batch correction, better use of metadata for differential comparisons, inclusion of trajectories analyses and simplified further analysis in future releases.

All workflow components now use Bioconda (18) packages, enabling tight versioning and automatic Docker container generation. For fastq utils, Scanpy and DropletUtils we contributed new wrapper scripts and Bioconda recipes to generate the necessary packages. These packages are then deployed using workflow infrastructure. We currently employ Nextflow at the quantification stage, and Galaxy (19) for downstream analysis in a reproducible way. See https://github.com/ebi-gene-expression-group/scxa-workflows for detail.

### User interface

The website can be searched by genes within or across species to reveal experiments, tissues and cell types where this gene is expressed or under which conditions it is a marker gene. Within each study, cells can be visualized using a pre-calculated t-SNE plot and can be coloured by different metadata or by cell clusters, based on gene expression. Gene expression across different cells is also displayed using a t-SNE-based visualization. Within each experiment, there are links to downloadable files, such as RNA quantification matrices, clustering results, reports on protocols and associated metadata, such as assigned cell types.

#### Gene search

**Figure 2.**
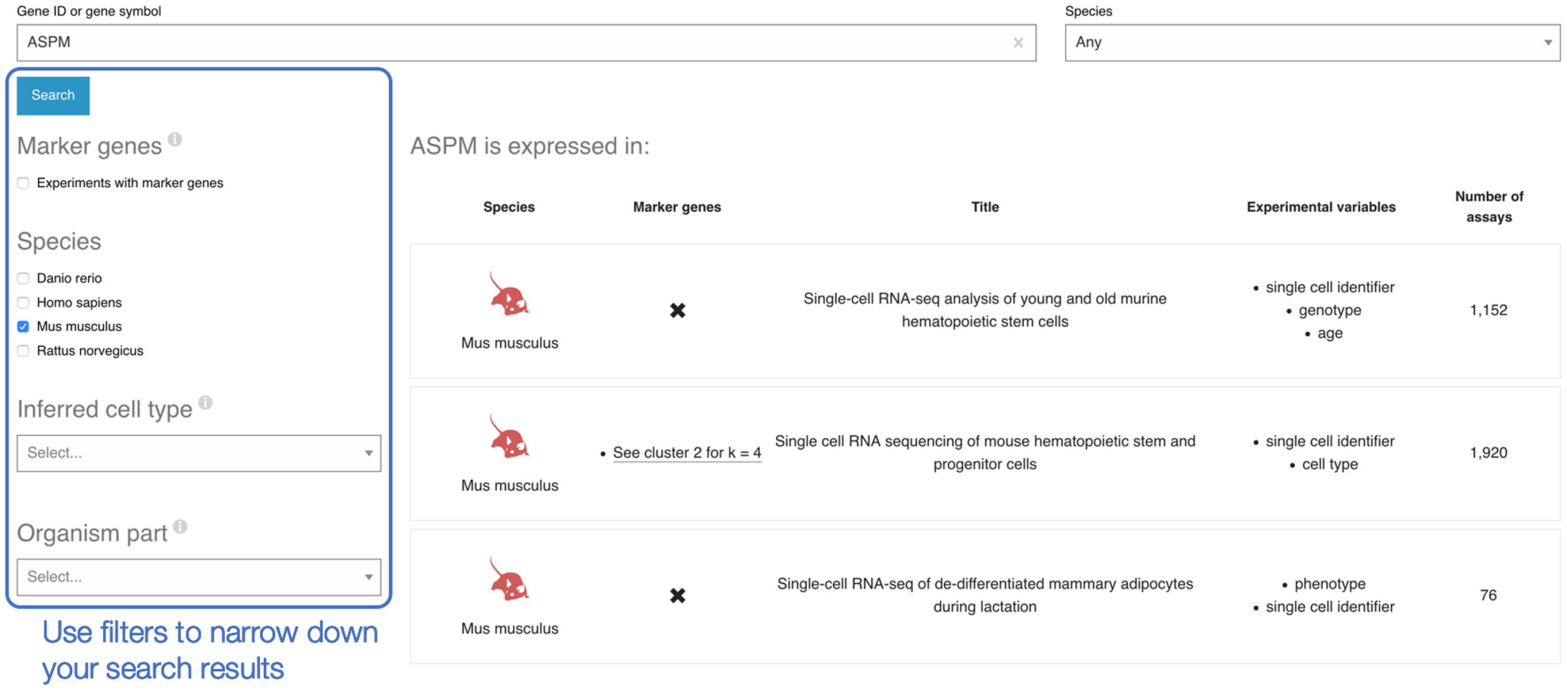
Search results for gene ASPM, filtered to show datasets where ASPM is expressed in *Mus musculus*.

Single Cell Expression Atlas home page includes queries by gene name and features, specifically query terms can be a gene ID, gene symbol or name. A species may be specified using a drop-down box next to the search box. Once a search is performed, the results will show all experiments where the gene is expressed. The results page shows a list of experiments from one or more species, depending on the query, indicating the species, the study title, experimental variables and number of assays. There is a clear indication whether the queried gene is a marker gene within the study. Filters on the left side of the page can be used to narrow down the search results as shown in Figure 2.

#### The experiment page

**Figure 3.**
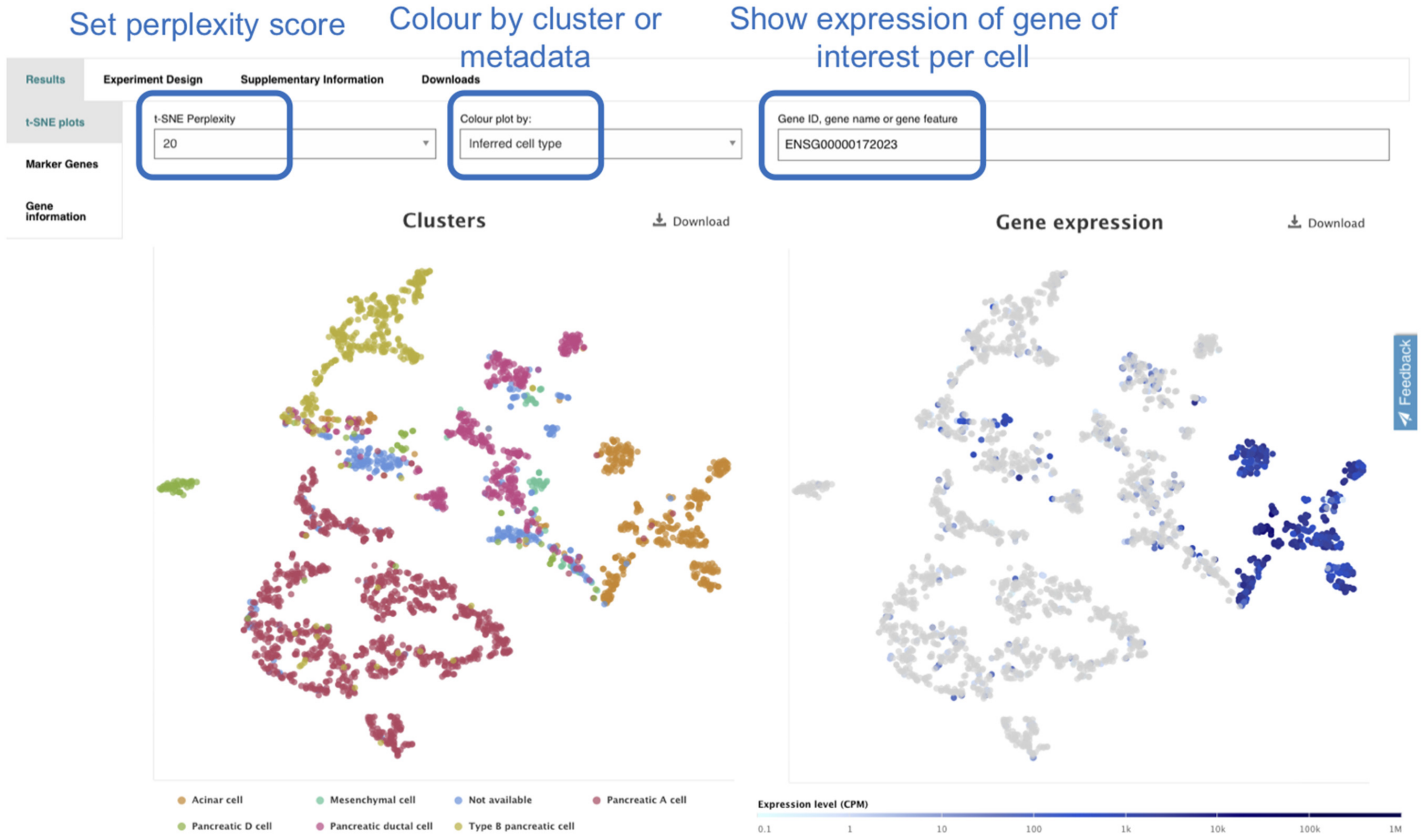
The experimental results of the ‘Single cell transcriptome of the human pancreas’ as shown in https://www.ebi.ac.uk/gxa/sc/experiments/E-GEOD-81547/results/tsne?geneId=ENSG00000172023&colourBy=metadata&metadata=inferred_cell_type The results are presented by two identical t-SNE plots where cells are coloured by ‘inferred cell type’ values on the left and the expression levels of gene REG1B (ENSG00000172023) on the right. By viewing these results side by side, a user can easily infer that REG1B is primarily expressed in pancreatic acinar cells.

**Figure 4.**
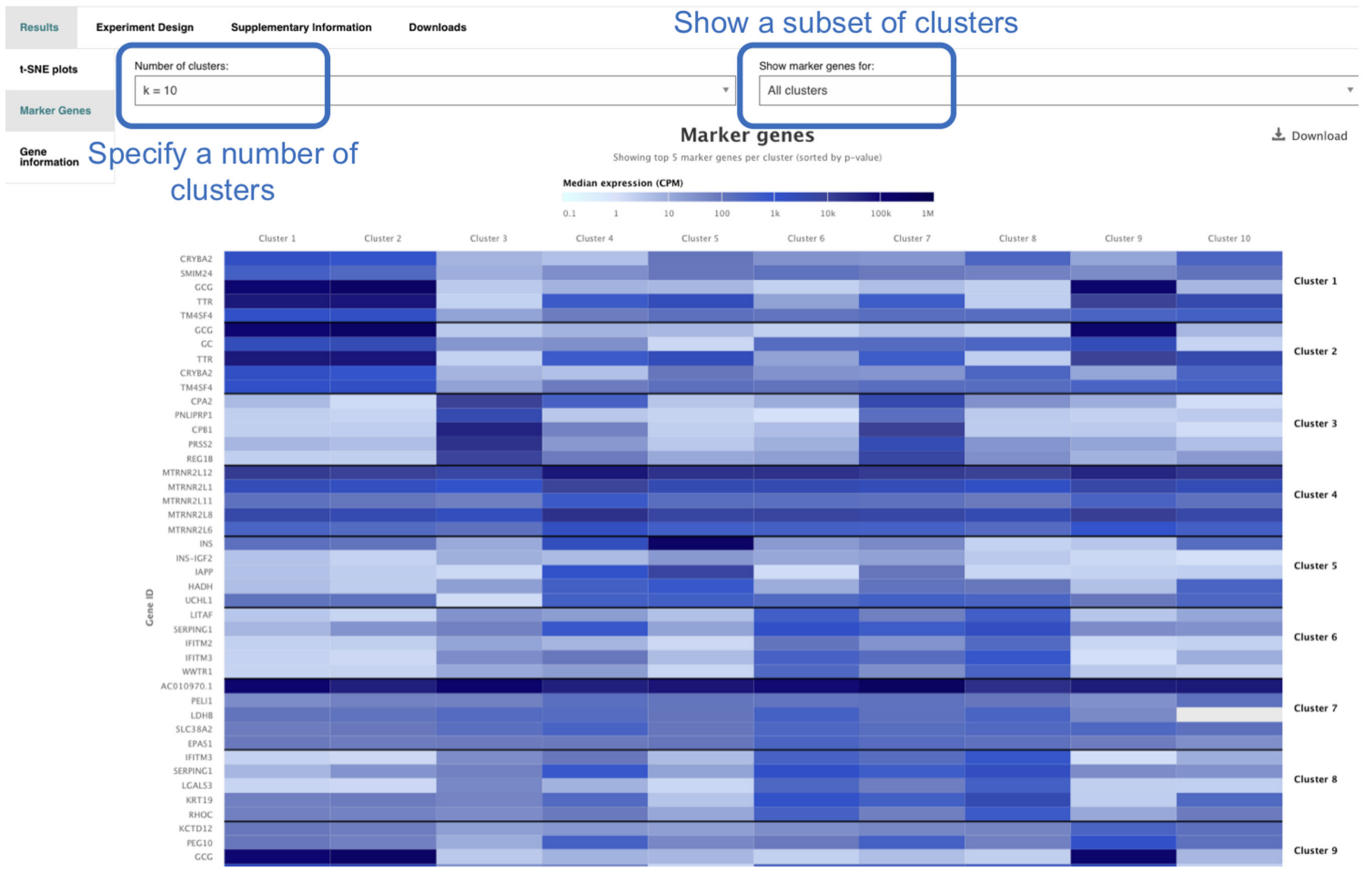
Screenshot of a heatmap showing the average expression of ‘marker genes’ within each cell cluster of a single study as shown in https://www.ebi.ac.uk/gxa/sc/experiments/E-GEOD-81547/results/tsne?geneId=ENSG00000172023&colourBy=metadata&metadata=inferred_cell_type&markerGeneK=10.

Each experiment in Single Cell Expression Atlas has its own experiment page that displays the results of the data analysis, provides links to the original archival source of the study, its publication and downloads of raw, processed and metadata files. The experiment page comprises of two visualization methods: t-SNE plots (Figure 3) and the marker gene heatmap (Figure 4). The t-SNE plots display subpopulations of cells within the data and at the same time enable the visualisation of gene expression variation at single-cell resolution.

#### Browse & download experiments

Finally, Single Cell Expression Atlas provides a page that shows an updated table of all available experiments. There are options to search the datasets by keywords, such as species or experiment title to filter the results, quick links to the analysis results for each experiment, but also an easy way to download data bundles from one or more experiments selected (Figure 1). The data bundles contain sample metadata files in MAGE-TAB format, as well as filtered and unfiltered normalised gene quantification matrices in matrix market format.

#### t-SNE widget

The t-SNE visualisation, with the included gene search, are available as a widget that can be embedded in different websites and resources wishing to link to the gene expression results of a particular study in Single Cell Expression Atlas. The widget, along with instructions on its usage are available here: https://github.com/ebi-gene-expression-group/scxa-tsne-widget.

## NEW DATASETS AND SPECIES

At the time of writing, Expression Atlas contains both bulk and single cell expression datasets as part of its newest component the Single Cell Expression Atlas. Collectively, these contain 3711 transcriptomic or proteomics studies (123 of which are single-cell), comprising a total of 1 070 052 assays. All Atlas datasets cover 62 species including 1381 studies on human, over 2600 studies on mammals and 810 studies in plants. Table 1 summarizes the top 15 species represented by the number of studies available in Expression Atlas. The datasets cover over 900 cell types from the Cell Ontology and over 963 diseases represented in EFO—the EMBL-EBI’s ontology for annotating functional genomics experiments (20). Although the majority of the datasets have been generated by the microarray technology (2758 studies), there are 809 studies based on bulk RNA sequencing and 21 proteomics datasets from human cancer cell lines and mouse. From the RNA-Seq datasets, 173 report baseline gene expression. Baseline data are now available for 42 species, with latest additions being beetroot (*Beta vulgaris*), rapeseed (*Brassica napus*), banana (*Musa acuminata*), peach (*Prunus persica*) and red clover (*Trifolium pratense*), amongst others. The majority of studies continue to be of differential design, consisting of 3391 datasets, studying samples in 9852 differential comparisons, in 47 different organisms.

**Table 1.** Fifteen most represented organisms in Expression Atlas—by the number of studies

Species	Number of differential studies	Number of baseline studies	Number of single cell studies
*Homo sapiens*	1282	46	53
*Mus musculus*	1023	42	50
*Arabidopsis thaliana*	535	7	5
*Rattus norvegicus*	142	3	1
*Drosophila melanogaster*	134	1	1
*Oryza sativa*	79	4	0
*Saccharomyces cerevisiae*	42	0	1
*Gallus gallus*	30	3	0
*Zea mays*	29	12	0
*Caenorhabditis elegans*	25	1	1
*Sus scrofa*	23	1	0
*Vitis vinifera*	16	4	0
*Danio rerio*	15	1	7
*Glycine max*	13	8	0
*Hordeum vulgare*	12	3	0
Others	140	55	1

Expression Atlas remains committed to making available and visualizing large-scale datasets for the benefit of the wider scientific community. These include version 6 of Genotype-Tissue Expression (GTEx) (21), Arabidopsis Information Portal version 11 (ARAPORT11) (22), FANTOM5 (https://www.ebi.ac.uk/gxa/experiments?experimentSet=FANTOM5); ENCODE (https://www.ebi.ac.uk/gxa/experiments?experimentSet=ENCODE) and proteomics data via the Human Protein Atlas. Landmark single cell datasets include the SMART-seq2 part of the datasets from the *Tabula muris* project (https://www.ebi.ac.uk/gxa/sc/experiments/E-ENAD-15/) and the Malaria Cell Atlas (https://www.ebi.ac.uk/gxa/sc/experiments/E-CURD-2/).

## OTHER DEVELOPMENTS

### Proteomics data

Since the last update, we significantly increased the content of proteomics datasets in Expression Atlas, in collaboration with the PRIDE team at EMBL-EBI. Expression Atlas now includes protein expression results from 21 mass spectrometry experiments on human cancer cell lines, cancer samples (https://doi.org/10.1101/665968) and different mouse tissues that have been analysed by a pipeline using MaxQuant as the base (6).

### User experience

Since the last update, we have implemented significant enhancements and new functionalities within the bulk Expression Atlas resource. Expression Atlas now displays transcript quantifications for many bulk RNA-Seq experiments using boxplots, and highlights the dominant transcripts where available. The autosuggestions on the search boxes have been improved and the search results are now displayed with a new “Filters" button that enables slicing the display according to the selected expression level. Moreover, proteomics and transcriptomics results are now more easily distinguishable. There has been a major redesign of the homepage, with the search bar now being on top, including information about the number of species, studies, assays, Ensembl/EFO versions for greater visibility.

## FUTURE DIRECTIONS

In the future, Expression Atlas will continue to import expression datasets across all species, analysing them uniformly and making them available freely and openly to the research community. Unlike GTEx, Araport, ENCODE and the Human Cell Atlas portals, which all are a project specific, the Expression Atlas integrated data from multiple datasets, including the ones from these projects, in a single interface and processed in a uniform way.

We will continue to develop Single Cell Expression Atlas and will focus on combining the precision of gene expression at the single cell level to the breadth of gene expression across a variety of conditions and tissues within the main components of Expression Atlas. For instance, we will implement automated methods for annotating cell types as derived from expression data. The infrastructure behind Expression Atlas will continue to scale up as data volumes increase and the interfaces will improve to enable queries on cell types. We will explore possibilities to provide a wider range of data visualization rather than t-SNE plots, including UMAP or PCA to name a few. It is possible that in the future the difference between the bulk and single-cell gene expression will be blurred, with many studies including both and with the wider use of micro-sample profiling. To address this challenge we will be working towards seamless integration of all Expression Atlas components into a single interface.

## Notes

Present address: Nuno A. Fonseca, CIBIO/InBIO, Centro de Investigação em Biodiversidade e Recursos Genéticos, Universidade do Porto, Portugal.

Present address: Cesar Augusto Prada Medina, Universidade de Sao Paulo (USP), Sao Paulo, Brazil.

Present address: Laura Huerta, DotmaticsLimited, Bishop's Stortford CM23 2ND, United Kingdom.
